# Federated learning with dynamic weighted aggregation for multi-crop disease detection: a hybrid CNN-transformer approach

**DOI:** 10.3389/frai.2026.1751118

**Published:** 2026-05-15

**Authors:** Ragoor Shashank, R. M. Bhavadharini

**Affiliations:** School of Computer Science and Engineering, Vellore Institute of Technology, Chennai, India

**Keywords:** agriculture, CNN-transformer, crop disease detection, dynamic aggregation, federated learning, machine learning

## Abstract

Crop diseases pose a significant threat to agriculture globally, causing a loss of 220 billion dollars annually. The problem can be solved through traditional machine learning methods which run on central servers but face two main obstacles: farmers refuse to provide their farming information and the uneven distribution of crop diseases across different regions. This paper introduces a federated learning framework that addresses these challenges through a novel approach that combines hybrid CNN-Transformer architectures with dynamic weighted aggregation. Our system applies EfficientNet-B0 and MobileNetV2 lightweight models which use MobileViT blocks together with CBAM and ESA attention mechanisms to extract detailed features from crop images. The innovation lies in the dynamic aggregation strategy, “AdaClass”-Adaptive Class-Aware aggregation, that identifies the underperforming classes in each round using class-wise F1 score and emphasizes the clients that are having a stronger performance on challenging disease classes.This approach helps in promoting a balanced performance across different disease classes. This particularly benefits the disease classes that are difficult to detect. Extensive experiments on two standard datasets demonstrate strong results: the proposed EfficientNet-B0 hybrid model achieves 99.32% accuracy on PlantVillage and 92.5% on CCMT datasets, while the MobileNetV2 achieves 99.17% and 91.4% respectively. Importantly, these models remain efficient enough for deployment on edge devices, with the MobileNetV2 hybrid requiring only 7.63 MB storage and processing images in 41.2 milliseconds.

## Introduction

Implementing AI based crop disease detection in real world agriculture has two major concerns: privacy and data heterogeneity. Farmers are not willing to share their raw images containing visual information across institutions, as it raises ownership and privacy concerns. Also at the same time, the occurrence of diseases varies based on the region, crop and climatic conditions resulting in highly non-IID(Not Independent and Identical Distribution) data distributions due to which only a limited number of subsets are observed at each farm. Federated Learning (FL) provides a good approach to solve this problem by training in a collaborative manner without the need for centralized data (McMahan et al., 2017). However the non-IID problem is amplified as the agricultural settings make balanced learning difficult for the model (Zhao et al., 2018).

The majority of FL systems use Federated Averaging (FedAvg), which combines client models proportionally to local dataset size (McMahan et al., 2017). Though it is straightforward and efficient for communication, this strategy indirectly favors the clients with larger datasets. Thus, uncommon or region-specific diseases play only a minor role in global updates. Federated Batch Normalization methods are one of them, reducing distribution shifts through localized normalization (Li et al., 2021), and multiple agricultural FL frameworks are proposed to integrate higher architectures such as transformers and hybrid models to enhance recognition accuracy (Dosovitskiy et al., 2020; Mehta and Rastegari, 2021). However, aggregation is still driven by the size of the data or based on the general performance of the client without considering the consistently underperforming disease classes.

Most FL methods don't actively pay attention to the classes the global model is consistently struggling with. They also fail to notice the clients which have strong expertise in identifying the challenging classes. Because of this, aggregation processes tend to favor the clients with larger datasets or common disease classes, while the challenging classes receive less attention.

To overcome this limitation, we propose the following federated aggregation framework as a targeted solution with the following contributions:

- **Class-percentile based struggling class detection:** Finding underperforming disease classes on a global scale through class-wise performance statistics.
- **Expertise-aware client weight amplification:** Adaptive aggregation to increase the influence of clients showing strong performance in challenging disease classes.
- **Cross-dataset generalization study:** Assessment of several agricultural datasets to ensure robustness under heterogeneous environments and each crop is evaluated independently inorder to isolate the data-specific heterogenity.

## Related work

###  Deep learning for crop disease detection

Deep learning models have become widely used methodology for recognizing and diagnosing crop diseases from visual data. Recent research works showed that Convolutional Neural networks (CNNs) can classify crop diseases directly from leaf images with high accuracy (He et al., 2016). CNN models like ResNet, EfficientNet and MobileNet made crop disease detection easier, leading to train deep learning models and capture more complex visual patterns. These CNN models can detect local texture changes, including spots, color shifts, and lesions which can indicate infection in the leaf images.

These Deep Learning models have been further improved by adding attention mechanisms such as Convolutional Block Attention Module (CBAM) which enhances feature representation in feature maps while reducing the influence of less relevant background areas (Woo et al., 2018). Further studies in agriculture depicted that attention-based CNNs shows good performance when crop disease symptoms are weak, small, or visually similar across classes (Gök et al., 2024; Yang et al., 2023). This is very important characteristic of attention mechanism for proper disease recognition, where classes differ only by subtle visual cues.

Standard CNNs can learn local patterns effectively rather than global context representations within an image (Howard et al., 2017, 2019). Transformer-based models address this issue by using self-attention to relate spatially seperated regions. The Vision Transformer (ViT) splits an image as a sequence of patches, similar to tokens in a sentence, and learns global relationships through attention mechanisms (Dosovitskiy et al., 2020). However, these transformers require large amount of training data and more computation power to learn feature representations than regular CNN models.

Several hybrid architectures have been proposed in the research studies to balance accuracy with efficiency. As discussed in Mehta and Rastegari (2021), MobileViT, for example, combines convolutional layers with lightweight transformer blocks to capture both local details and global context in the image. Edge devices with limited resources can use these models more effectively. Research studies in Mamba Kabala et al. (2023), Fahim-Ul-Islam et al. (2024), and Sharma et al. (2024) deployed hybrid CNN–Transformer models for multi-crop disease detection and disease severity estimation, and reported better generalization across crops and environments. Despite their performance increase, these models are still mostly trained in a centralized controlled environment, where all data are uploaded to one server.

###  Federated learning in agriculture

Federated learning (FL) is a distributed environment, where multiple clients collaborate on a shared model keeping data localized without sending raw data to a central server (McMahan et al., 2017). Each client trains the model locally on its own data and then transmits only the updated parameters to a central server that aggregates them into a new global model. This approach is well suited for agriculture applications, where farm-level data may have sensitive information about crop management practices, crop health, and estimated yields.

Federated Averaging (FedAvg) is the most commonly used Federated Learning aggregation technique. In FedAvg, the global model parameters are updated using weighted average of client models, where the weight of each client is proportional to the size of its local dataset.

The performance of FedAvg degrades when data distributions differ across clients (Choubey and Divya, 2024; Hari and Singh, 2025). Such non-IID conditions are common and reasonable in agriculture farms because crop diseases differ across regions, climate conditions, and crop varieties.

Several methods and models have been proposed to cope with this heterogeneity in the dataset. FedBN is a FL algorithm designed to deal with feature distribution heterogeneity across clients by retaining batch normalization parameters locally (Li et al., 2021). A number of other existing FL approaches modify aggregation weights based on models' validation performance instead of relying only on dataset size (Li T. et al., 2020; Karimireddy et al., 2020). These strategies improve training stability but mainly optimize global accuracy.

Research studies have been carried out for communication-efficient FL designs under resource-limited rural environments. Lightweight federated transfer learning aims to reduce computational cost while retaining acceptable accuracy (Choubey and Divya, 2024). Client selection strategies based on entropy or participation scheduling are better performing under limited bandwidth and unstable connectivity conditions (Lai et al., 2021; Herath et al., 2024). More recent work combines transformer-based vision models with FL for distributed crop disease detection (Mamba Kabala et al., 2023). Compared to traditional Convolutional Federated models, these approaches typically achieve higher recognition performance.

Although there is a significant performance improvement in the global model's overall accuracy, these models rarely examine which disease classes are consistently misclassified across clients. Consequently, rare or region-specific diseases may remain underrepresented during aggregation process in FL. Addressing this gap requires methods that incorporate class-level performance into the aggregation process, rather than depending only on data volume or overall accuracy.

Tables 1, 2 jointly highlight two important observations. First, although federated learning has been increasingly applied to crop disease detection, most studies in the literature survey use standard aggregation schemes such as FedAvg or minor variants focused on convergence stability. These methods rely on client weight updates based primarily on dataset size or optimization regularization, without incorporating explicit individual class-level performance information. As a result, rare or region-specific diseases may go underrepresented during global model updates.

**Table 1:** Comparison of federated learning aggregation approaches.

Method	Year	Class-aware	Adaptive	Approach
FedAvg (McMahan et al., 2017)	2017	No	No	Data-proportional weights
FedProx (Li T. et al., 2020)	2020	No	No	Proximal regularization
SCAFFOLD (Karimireddy et al., 2020)	2020	No	Yes	Control variates
FedNova (Wang et al., 2020)	2020	No	Yes	Normalized averaging
FedDyn (Acar et al., 2021)	2021	No	Yes	Dynamic regularizer
**AdaClass (Proposed)**	2025	Yes	Yes	Class-targeted expertise

*Class-Aware*: Explicitly models per-class performance. *Adaptive*: Dynamically adjusts aggregation weights.

**Table 2:** Comparison of recent federated and deep learning approaches for crop disease detection (2023–2025).

Author	Year	Dataset	Model / Method	Metrics	Limitations
Kabala et al.	2023	PlantVillage (38 classes)	FL with CNNs (ResNet50, MobileNetV2, ViT) using FedAvg	Accuracy, Training Time	Sensitive to FL hyperparameters; ViT computationally heavy
Aggarwal et al.	2023	Rice dataset (5,932 images)	Lightweight FL with EfficientNetB3 + Dense classifier	Accuracy (97%–99%)	Performance drops under strong non-IID conditions
Islam et al.	2024	Wheat disease (Kaggle datasets)	CoAtNet + Swin Transformer with FedMax aggregation	Accuracy, Precision, Recall, F1	Dependent on data diversity; pruning may reduce features
Choubey and Divya	2024	Multi-client rice dataset	Lightweight Federated Transfer Learning (LFTL)	Accuracy, Privacy Score	Struggles with class imbalance and large disease variety
Herath et al.	2025	MNIST, CIFAR-10/100	Dynamic Data Queue FL (Entropy-based weighting)	Accuracy, Convergence Rate	Not agriculture-specific
Chorney et al.	2025	Multi-site rice dataset (10,553 images)	FedAvg, q-FedAvg, PT-FedAvg	Accuracy	Poor performance for unique-disease clients
Hari and Singh	2025	PST (50k), BCG (40k) datasets	Intelligent Weight Transfer (FDL-IWT) with Dirichlet weighting	Accuracy (95%–97%)	Device heterogeneity; noisy images
Li et al.	2025	MetaFruit, Harmful Animal datasets	VLLFL (Vision-Language FL) with prompt tuning	mAP, Communication Cost	Limited by base VLM knowledge
Zubair et al. (2025)	2025	PlantVillage, PlantDoc, FieldPlant	Ensemble CNN (InceptionResNetV2, MobileNetV2, EfficientNetB3)	Accuracy, F1	High computational cost; no privacy handling
Yang et al.	2023	AI Challenger 2018	Triple-branch Swin Transformer (centralized)	Accuracy (99%), F1	Severity confusion; centralized training only

Second, while hybrid CNN–Transformer architectures have shown strong performance under centralized crop disease classification environment, their integration with heterogeneity-aware federated aggregation remains limited. Most of the existing federated approaches in Table 2 primarily optimize global model accuracy and communication efficiency, but do not explicitly address the interaction between non-IID class distributions and client expertise.

In contrast, the proposed research work introduces a class-aware adaptive aggregation mechanism, AdaClass that identifies globally underperforming disease categories and dynamically adjusts weights from clients demonstrating expertise in those classes. By combining edge-efficient hybrid modeling with targeted aggregation under non-IID conditions, the method addresses both deployment constraints and class imbalance challenges in distributed agricultural environments.

## Methodology

Our approach addresses multi-crop disease detection through a federated learning system. This work evaluates the proposed FL aggregation method, AdaClass, independently across both Maize and Tomato crop datasets—demonstrating a consistent behavior under non-IID conditions. This keeps farmer data private while enabling collaborative model improvement. The system works by training lightweight hybrid models on individual farms and then combining their knowledge through a smart aggregation strategy.

###  Problem formulation

We consider a federated learning scenario with *K* clients (farms), each holding a local dataset $D_{i}={\left\{\left(x_{j},y_{j}\right)\right\}}_{j=1}^{n_{i}}$ of crop disease images and labels *y*_*j*_ ∈ {1, …, *C*}. The clients collaboratively train a global model θ by aggregating local updates without sharing raw data.

#### Objective

Find optimal parameters θ^*^ minimizing:


$$\begin{array}{l} \theta^{*}=\arg\underset{\theta }{\min}\overset{K}{\underset{i=1}{\sum }}w_{i}L_{i}\left(\theta \right) \end{array}$$ (1)

subject to: $\overset{K}{\underset{i=1}{\sum }}w_{i}=1$ and *w*_*i*_ ≥ *w*_*min*_.

#### Challenge

Majority of the agricultural data is heterogeneous (non-IID) with different diseases types present across various regions. FedAvg uses fixed weights $w_{i}=\frac{n_{i}}{\underset{j}{\sum }n_{j}}$ which is completely based on the size of the dataset, ignoring that some clients or participants may perform better in specific diseases.

#### Our solution

AdaClass dynamically computes aggregation weights by:

1. Using confidence weighted F1-score to identify the disease classes on which the global model is underperforming: ${\bar{F}}^{\left(c\right)}\leftarrow \overset{K}{\underset{i=1}{\sum }}\frac{n_{i}}{N}F_{i}^{\left(c\right)}$ for each class *c*, where $N=\underset{j}{\sum }n_{j}$.
2. Prioritizing the clients who excel at the specific classes on which the global model is underperforming.
3. Balancing data-proportional and performance-based weighting.

The complete adaptive weight computation is detailed in Algorithm 1.

Algorithm 1 AdaClass: adaptive class aware aggregation. 
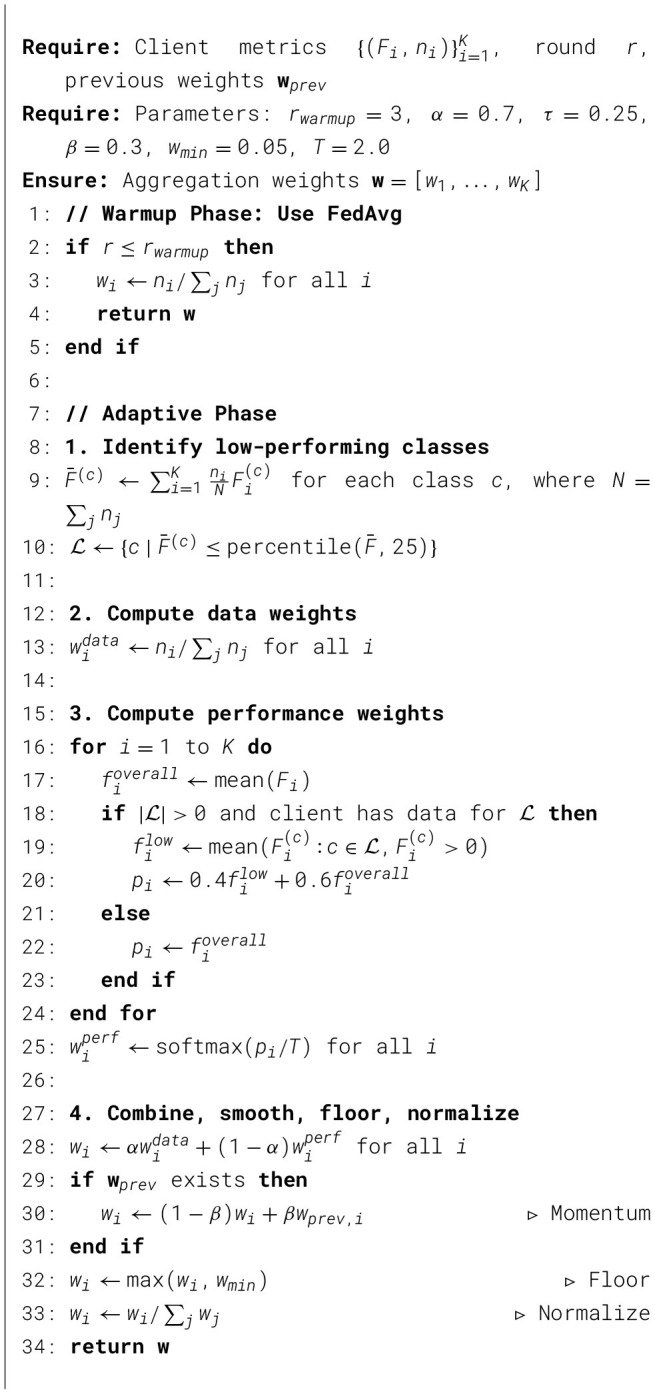

###  Datasets and experimental setup

#### PlantVillage

The PlantVillage, a publicly available dataset contains 54,305 lab captured images with 38 disease classes across 14 crops with a homogenous background. This dataset shows moderate class imbalance across different categories (Hughes and Salathe, 2015).

#### CCMT dataset (maize subset)

The CCMT, a publicly available dataset contains 24,881 field captured images from the land of Ghanna is a real world agricultural benchmark across four crops (cashew, cassava, maize, and tomato). In this study, we focus on both maize and tomato subset, containing 5,358 and 5,805 original images with seven and 5 disease classes respectively (Kwabena et al., 2023).

During training, augmented samples provided with the dataset are incorporated to improve generalization as well as reduce class imbalance. The final maize dataset after augmentation and directory consolidation consists of 24,551 images (19,426 training, 5,125 testing), similarly tomato subset also contains 27,168 images (21,723 training and 5,445 testing).

#### Class distribution and imbalance handling

The maize subset shows distinct class imbalance as in Table 3. Minorities, including *Healthy* (4.24%) and *Fall Armyworm* (5.80%) are under-represented compared to dominant classes like *Leaf Blight* (20.48%) and *Streak Virus* (20.56%). Similarly, the tomato class also shows class imabalance. Minority classes which are under-represented include *Healthy* (9.10%) and *Leaf Curl* (9.39%). Dominant classes include *Septoria Leaf Spot* (42.60%) and *Leaf Blight* (23.68%)

**Table 3:** CCMT dataset: train–test class distribution for maize and tomato subsets.

Disease class	Train	Test	Total	Prop. (%)
Maize (7 classes, 10 clients)				
Fall armyworm	1,140	284	1,424	5.80
Grasshopper	2,575	411	2,986	12.16
Healthy	830	211	1,041	4.24
Leaf beetle	3,789	950	4,739	19.30
Leaf blight	4,025	1,004	5,029	20.48
Leaf spot	3,024	1,261	4,285	17.45
Streak virus	4,043	1,004	5,047	20.56
**Total**	**19,426**	**5,125**	**24,551**	**100.00**
Tomato (5 classes, 10 clients)				
Healthy	2,000	500	2,500	9.10
Leaf blight	5,200	1,309	6,509	23.68
Leaf curl	2,050	532	2,582	9.39
Septoria leaf spot	9,373	2,340	11,713	42.60
Verticillium wilt	3,100	764	3,864	14.06
**Total**	**21,723**	**5,445**	**27,168**	**100.00**

Data based on local directory analysis of the CCMT subset. Both subsets exhibit class imbalance; Septoria Leaf Spot dominates the Tomato subset (42.60%) while Maize shows a more even spread across seven classes with Healthy as the minority (4.24%).

To compensate for the imbalance, two complementary measures are implemented:

**(1) Online data augmentation** A combination of random horizontal and vertical flipping; rotation (±15°), affine transformations, color jitter, CLAHE based contrast enhancement, and Gaussian/bilateral denoising are dynamically used in the training phase in order to improve the data diversity and robustness.
**(2) Class-weighted loss** Class weights are obtained from the global training distribution using a balanced weighting equation

$$w_{c}=\frac{N}{K\cdot n_{c}}$$

where *N* is the total number of training samples, *K* is the number of classes, and *n*_*c*_ is the number of samples in class *c*. These weights are included in a weighted cross-entropy loss, raising the penalty of minority-class misclassifications.

#### Federated learning setup

To simulate distributed agricultural environments we experimented with two setups: **3-client setup:** The experimentation is done on three clients with Dirichlet distribution with concentration parameter α = 0.3 to validate proposed architecture and algorithm under controlled with reduced heterogeneity of disease classes among clients, leading to isolation of the model architecture contributions from federated complexity.

**10-client setup:** The training data is divided into ten virtual clients utilizing Dirichlet distribution with concentration parameter α = 1.0, creating moderate to high statistical diversity across clients. The client-wise distribution can be summarized in Table 4. Mean Jensen-Shannon (JS) divergence is 0.490 ± 0.130, leading to a marked deviation from uniform label distribution and realistic non-IID behavior.

**Table 4:** CCMT client data distribution (dirichlet α = 1.0)—maize and tomato.

Client	Samples	Dominant class (%)	JS Div.	# Classes
Maize (7 classes)				
C0	2,040	Leaf spot (41.4)	0.352	5
C1	1,832	Leaf beetle (44.2)	0.428	7
C2	2,472	Streak virus (49.8)	0.556	4
C3	1,202	Streak virus (34.9)	0.385	5
C4	735	Leaf beetle (30.7)	0.278	5
C5	1,978	Leaf spot (40.4)	0.412	4
C6	2,346	Leaf beetle (55.2)	0.623	2
C7	1,717	Streak virus (27.5)	0.318	5
C8	1,898	Streak virus (73.8)	0.764	4
C9	3,206	Leaf blight (68.6)	0.687	4
**Mean**	**2,043**	**—**	**0.490**	**4.5**
**Std**	**707**	**—**	**0.130**	**1.3**
Tomato (5 classes)				
C0	2,383	Septoria L.S. (67.3)	0.214	3
C1	3,054	Leaf blight (53.2)	0.213	3
C2	2,046	Septoria L.S. (48.8)	0.052	5
C3	2,033	Septoria L.S. (42.6)	0.138	4
C4	2,956	Septoria L.S. (72.0)	0.289	2
C5	1,971	Vert. Wilt (46.3)	0.047	5
C6	1,930	Leaf blight (65.3)	0.281	2
C7	1,352	Leaf blight (39.3)	0.098	4
C8	2,546	Septoria L.S. (40.8)	0.108	4
C9	886	Septoria L.S. (37.5)	0.025	5
**Mean**	**2,116**	**—**	**0.147**	**3.7**
**Std**	**633**	**—**	**0.092**	**1.1**

*JS Div*.: Jensen-Shannon divergence from uniform distribution. Values > 0.35 indicate high heterogeneity. Maize exhibits stronger non-IID behavior (mean JS = 0.490) than Tomato (mean JS = 0.147), providing complementary heterogeneity conditions for evaluating cross-crop generalization of AdaClass.

All 10 clients are participated in every round, each round consists of 2 epochs with a total of 18 rounds. Similarly, in 3-client setup, all the 3 clients participate; each round consists of 1 epoch with a total of 30 rounds. In each round, the client trains the model locally using the AdamW optimizer, a learning rate of 0.001, a weight decay of 0.01 and with a batch size of 32 and 64, respectively in training and validation. Warm-up schedule including step decay is used to stabilize convergence.

Regularization mechanisms including online augmentation, class-weighted loss, dropout (0.2), weight decay, gradient clipping (max norm = 1.0), and learning rate scheduling are employed to minimize overfitting on smaller or highly skewed clients.

FedProx proximal term μ = 0.01, selected from the standard search range {0.001, 0.01, 0.1} recommended by Li X. et al. (2020); FedDyn regularization coefficient α_*dyn*_ = 0.01, drawn from the search space {0.1, 0.01, 0.001} defined in Acar et al. (2021). Both values were fixed across all seeds without further tuning.

##### Model selection

The global model from the final communication round (round 18 for 10 client setup and round 30 for 3-client setup) is chosen for evaluation. No early stopping is applied.

##### Random seeds

The random seeds {0,7,42,99,123} and {0,7,123} were chosen for maize and tomato respectively. These were applied consistently to torch.manual_seed, numpy.random.seed, random.seed, and the Dirichlet client-partition generator. CUDA determinism is enforced via

torch.backends.cudnn.deterministic = True and torch.backends.cudnn.benchmark = False. The Dirichlet partition is regenerated from the same seed for every method. This setup makes sure that evaluation for all aggregation methods is done under identical data distributions and training conditions across the datasets. This enables a fair comparison for all the aggregation methods.

###  Image preprocessing and augmentation

To improve image quality and model robustness, we distinguish between two complementary strategies applied at different stages of the training pipeline.

Preprocessing refers to a set of deterministic operations that is applied consistently to all images during both training and inference. CLAHE is applied to enhance local contrast between healthy and diseased tissue. Gamma correction (γ = 1.2) brightens images to reveal subtle disease symptoms in dim field conditions. Noise reduction using bilateral filtering or Gaussian blur is applied to mitigate sensor noise and compression artifacts.

**Figure 1:**
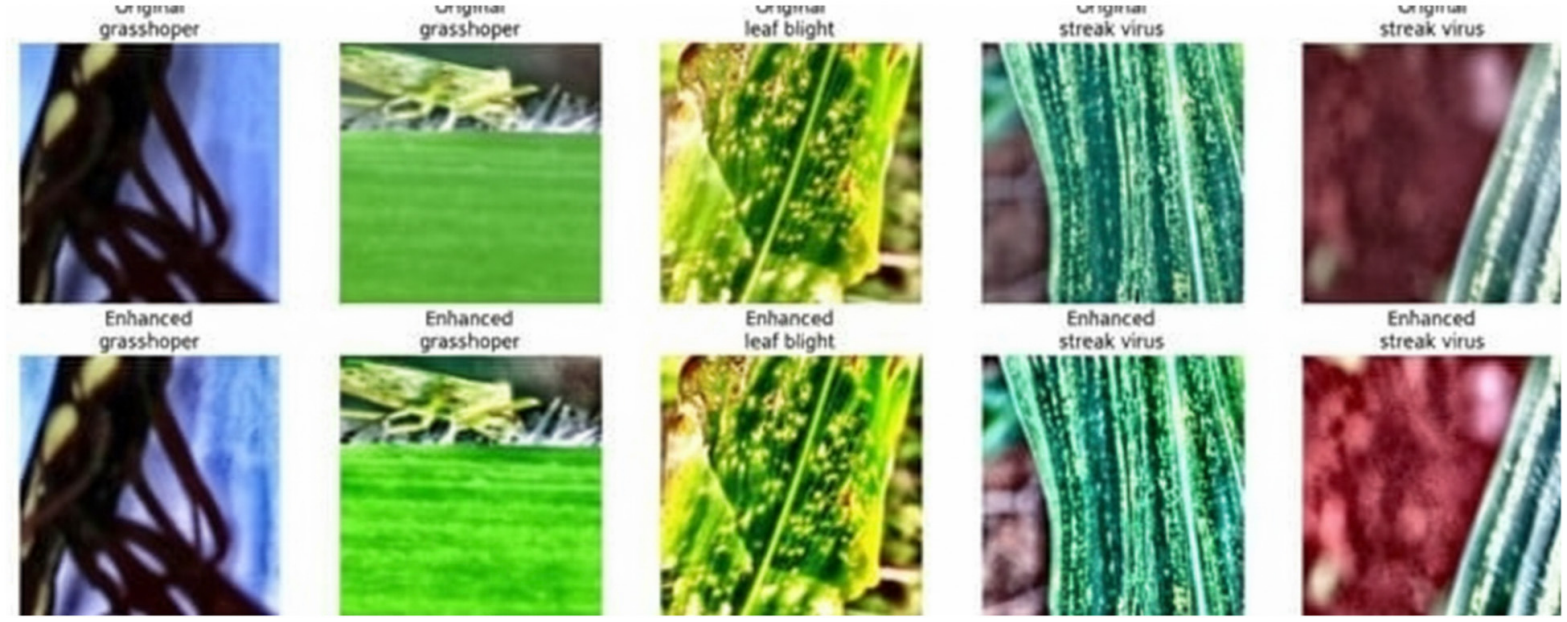
Image enhancement comparison before and after preprocessing.

Data Augmentation refers to stochastic transformations applied exclusively during training to increase data diversity and reduce overfitting. These include random horizontal and vertical flipping, rotation (±15°), affine transformations, color jitter, and edge sharpening (strength = 1.5), each applied with 50% probability per sample. These operations are disabled during inference to ensure consistent evaluation conditions. The results of the pipeline can be clearly seen in Figure 1 which shows the image preprocessing and augmentation comparison before and after processing.

### Hybrid CNN-transformer architecture

The proposed hybrid architecture merges the efficiency of CNNs with the global modeling capacity of transformers, principally applied to detect agricultural diseases.

#### Backbone networks

**Figure 2:**
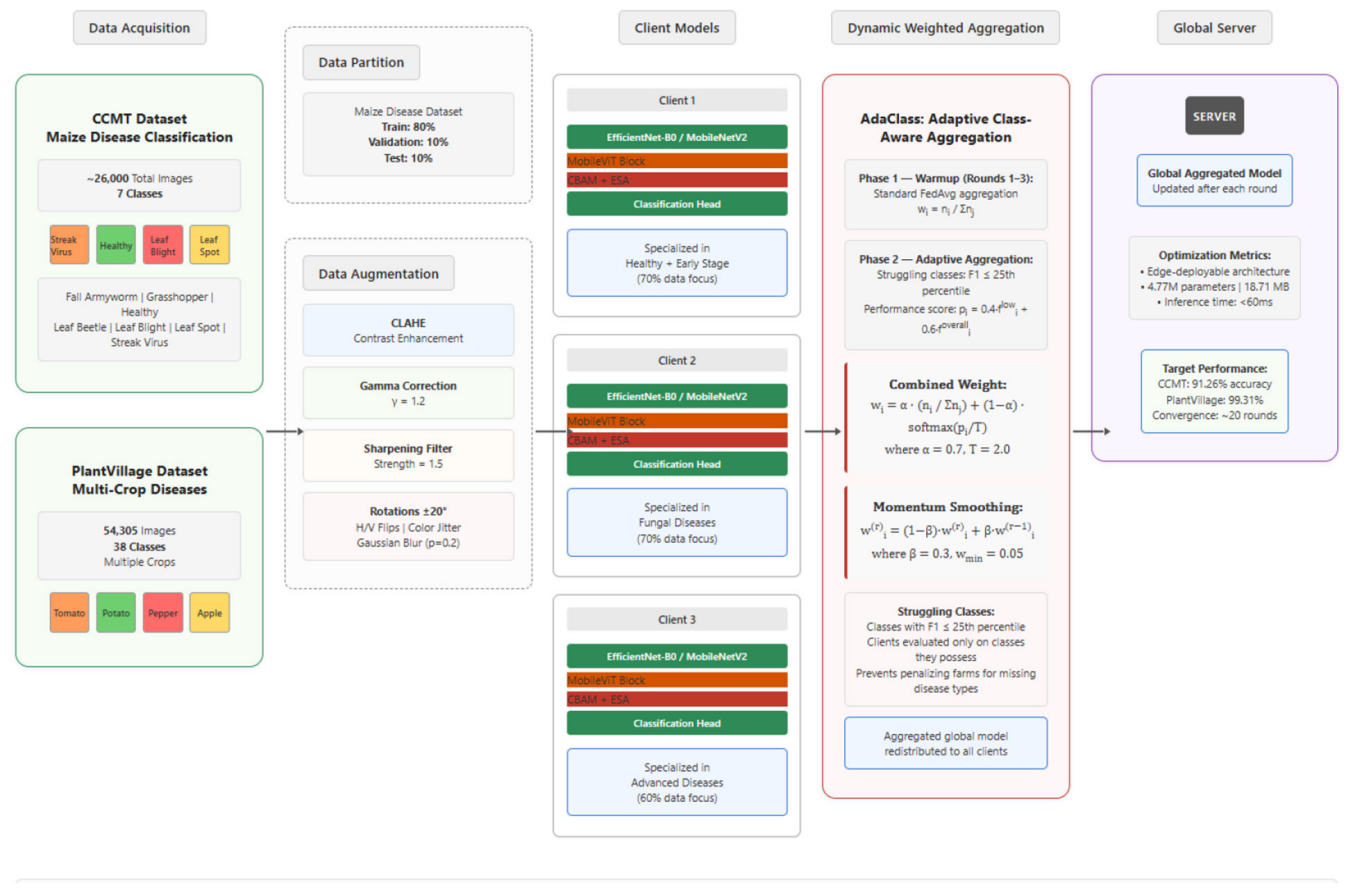
Proposed federated learning hybrid CNN-transformer model architecture for crop disease detection.

The Figure 2 shows two superclass backbones that were used in the 3-Client setup: EfficientNet-B0 (Tan and Le, 2019), which offers the best trade off between accuracy and efficiency (4.9M parameters, ~20MB) by compound scaling of depth, width, and resolution; and MobileNetV2 (Sandler et al., 2018) (3.2M parameters, 15MB), which allows lightweight deployment through inverted residual blocks and linear bottlenecks.

#### MobileViT integration

Following the architecture in Figure 2, MobileViT blocks are integrated by combining local convolution with global transformer attention mechanisms. This integration helps to extract local features through 3 × 3 convolutions, then they are converted into patches with the help of an unfold operation with patch size of 2. These patches undergo multi-head attention processing to capture global dependencies, followed by reshaping and 1 × 1 convolution operations.

1. **Local feature extraction:** First, local features are extracted from the input using a standard 3 × 3 convolution.
$$\text{Local\_Features}=\text{Con}{\text{v}}_{3\times 3}\left(\text{Input}\right)$$
2. **Patch creation:** The resulting local feature maps are then unfolded into a sequence of flattened patches, each with a size of 2.
Patches=Unfold(Local_Features,patch_size=2)
3. **Global feature modeling:** Multi-head attention is applied to these patches to capture the global dependencies and relationships across the entire image.
Global_Features=MultiHeadAttention(Patches)
4. **Final output generation:** Finally, the globally processed features are reshaped and passed through a 1 × 1 convolution. A residual connection is added from the original input to form the final output of the block.
$$\text{Output}=\text{Con}{\text{v}}_{1\times 1}\left(\text{Reshape}\left(\text{Global\_Features}\right)\right)+\text{Input}$$

#### Attention and classification

The architecture includes two attention modules, namely, CBAM and ESA. CBAM processes the MobileViT output through sequential channel and spatial attention. The enhanced features undergo global average pooling, with dropout (0.2), and linear classification as shown in Figure 2. Here in, Channel Attention focuses to learn the most discriminative features, and Spatial Attention learns to emphasize the most informative region in the feature maps. The ESA module is complementary to CBAM and further strengthens spatial awareness by performing a combination of average and max pooling operations, providing stronger spatial modeling. Besides, ESA exploits dilated convolutions to carry out multi-scale feature extraction that benefits fine-grained and contextual spatial information capture.

#### Model architectures

A hybrid CNN-Transformer design was used in this work, and the reasoning behind this choice is straightforward. CNNs generally are good at detecting the local patterns like lesion edges and texture, but they cannot easily reason about what is happening across the full leaf. Transformers can do that, but they are heavy and need a lot of data. So neither one on its own is a good fit for crop disease detection in a federated setting where data and compute are both limited.

First, MobileViT was brought in to bridge this gap cause it gives the same reasoning as the state of the art transformers with half the computational cost. Unlike a standard transformer that discards spatial structure, MobileViT keeps the local feature maps intact through convolution and then runs self-attention on top of that. This enables the model to capture the fine-grained details of a lesion and the broader spatial context across the leaf image. That combination turns out to be more useful for disease classification.

Moving on to the backbone selection, EfficientNet-B0 architecture summarized in Table 5 was chosen because it scales depth, width and resolution together in a controlled way rather than just making the network deeper or wider arbitrarily. As it moves through its seven MBConv stages, the spatial resolution drops from 112 × 112 down to 7 × 7 while the channel depth grows from 16 all the way to 320, and the head convolution then brings this up to 1280 channels before the features reach the MobileViT block. By the time that happens, the features are compact enough for attention to be applied efficiently but rich enough to carry strong semantic meaning. With 4.77M parameters, the model gives a good accuracy.

**Table 5:** EfficientNet-B0 + MobileViT backbone layer-wise architecture.

Stage	Layer name/type	Output shape	Params	Description
Input	input_225 (InputLayer)	(None, 225, 225, 3)	0	Input image
Stem	stem_conv + BN + Swish	(None, 32, 112, 112)	928	Initial feature extraction
MBConv1	block1a (MBConvBlock × 1)	(None, 16, 112, 112)	1,616	Mobile bottleneck block
MBConv2	block2a–2b (MBConvBlock × 2)	(None, 24, 56, 56)	13,084	Feature expansion stage
MBConv3	block3a–3b (MBConvBlock × 2)	(None, 40, 28, 28)	46,640	Expanded receptive field
MBConv4	block4a–4c (MBConvBlock × 3)	(None, 80, 14, 14)	242,930	Deep feature extraction
MBConv5	block5a–5c (MBConvBlock × 3)	(None, 112, 14, 14)	543,148	Feature enrichment
MBConv6	block6a–6d (MBConvBlock × 4)	(None, 192, 7, 7)	2,026,348	Context modeling
MBConv7	block7a (MBConvBlock × 1)	(None, 320, 7, 7)	717,232	Final CNN stage
Head	top_conv + BN + Swish	(None, 1,280, 7, 7)	412,160	Feature head convolution
MobileViT Block	Transformer + Conv2d	(None, 1,280, 7, 7)	547,344	Global context modeling
Attention Modules	Lightweight CBAM + ESA	(None, 1,280, 7, 7)	204,900	Feature refinement
Classification	Pool + Dropout + Linear	(None, 7)	8,967	Disease classification
**Total parameters**			**4,774,167**	

MobileNetV2 summarized in Table 6 was added as a second backbone that works differently from EfficientNet, using inverted residual blocks with linear bottlenecks and ReLU6 instead of compound scaling. This model arrives at the same 1,280-channel feature space through its head convolution layer. This shows that the MobileViT block and attention modules positioned identically in both models. The difference is that MobileNetV2 gets similar feature representation with only 1.55M parameters in total, roughly one third of Model A. This makes MobileNetV2 a genuinely practical option for edge deployment in the field.

**Table 6:** MobileNetV2 + MobileViT backbone layer-wise architecture.

Stage	Layer name/type	Output shape	Params	Description
Input	input_224 (InputLayer)	(None, 224, 224, 3)	0	Input image
Stem	stem_conv + BN + ReLU6	(None, 32, 112, 112)	864	Initial convolution
Bottleneck1	inverted_residual_1 ( × 1)	(None, 16, 112, 112)	896	Inverted residual block
Bottleneck2	inverted_residual_2 ( × 2)	(None, 24, 56, 56)	5,136	Expansion ratio 6
Bottleneck3	inverted_residual_3 ( × 3)	(None, 32, 28, 28)	8,832	Feature downsampling
Bottleneck4	inverted_residual_4 ( × 4)	(None, 64, 14, 14)	34,816	Deep feature maps
Bottleneck5	inverted_residual_5 ( × 3)	(None, 96, 14, 14)	54,272	High-dimensional features
Bottleneck6	inverted_residual_6 ( × 3)	(None, 160, 7, 7)	118,080	Spatial compression
Bottleneck7	inverted_residual_7 ( × 1)	(None, 320, 7, 7)	155,264	Final bottleneck
Head	conv_1x1 + BN + ReLU6	(None, 1,280, 7, 7)	409,600	Feature head
MobileViT Block	Transformer + Conv2d	(None, 1,280, 7, 7)	547,344	Global attention
Attention Modules	Lightweight CBAM + ESA	(None, 1,280, 7, 7)	204,900	Spatial/channel attention
Classification	Pool + Dropout + Linear	(None, 7)	8,967	Disease prediction
**Total parameters**			**1,548,971**	

Finally, CBAM and ESA were added to address non-uniform distribution of disease symptoms across a leaf. A rust patch or a blight spot may spread over a very small area in the leaf, and it may be missed out without attention mechanisms. CBAM handles this by filtering out uninformative channels and background regions step by step, and refining feature representations through channel and spatial attention. ESA then adds another layer of spatial awareness using dilated convolutions, which helps the model to identify difference in symptoms of the same disease under different levels of disease severity or lighting conditions. Together they add only 204,900 additional parameters, which results in minimal computational overhead compared to the performance improvement.

### System environment

All simulations were run on Google Colaboratory and Kaggle Notebooks. The Colaboratory environment provided an Intel Xeon CPU with 13 GB of RAM and an NVIDIA Tesla T4 GPU with 16 GB of VRAM. Kaggle Notebooks offered an Intel Xeon CPU, up to 30 GB of RAM, and either an NVIDIA Tesla T4 GPU with 16 GB of VRAM. Both platforms use a Linux-based Python environment.

###  AdaClass: adaptive class-aware aggregation

The AdaClass algorithm (Algorithm 1) dynamically modifies input of each client in the aggregation process with respect to two complementary factors: (i) the quantity of the local training data in the database of the client and (ii) whether each class of disease can be correctly classified by the client, which is currently the challenging to the global model.

The algorithm works in two phases:

#### Phase 1 (Warm-up)

Standard FedAvg aggregation is performed across all initial *r*_*warmup*_ rounds. At early train times, performance measures are volatile, due to the fact that models are still learning the most basic feature descriptions. The use of adaptive weighting here may amplify noisy signals and result in unreliable aggregation decisions. This warm-up phase guarantees stable estimation of metrics leading up to the start of adaptive reweighting.

#### Phase 2 (Adaptive aggregation)

After a model can stabilize, aggregation weights are increased dynamically to make sure that clients which perform well for globally underperforming disease classes will be selected as most important.

The algorithm goes through the works in a step-by-step manner.

#### Step 1: Identification of underperforming classes

After every communication round, the average F1-score for each disease class is calculated in all the clients *K*. Let $F_{i}^{\left(c\right)}$ denote the F1-score achieved by client *i* on class *c*, *n*_*i*_ the number of training samples at client *i*, and $N=\overset{K}{\underset{j=1}{\sum }}n_{j}$ the total sample count. The confidence-weighted global F1-score for class *c* is:


$$\begin{array}{l} {\bar{F}}^{\left(c\right)}=\overset{K}{\underset{i=1}{\sum }}\frac{n_{i}}{N}F_{i}^{\left(c\right)}. \end{array}$$ (2)

When weighted based on the proportion of the data, it makes sure that larger and reliable clients contribute more to the global class-level estimate. This reduces noise from clients with sparse data whose per class F1 estimates may be unstable. Classes are identified as underperforming when the average performance falls within the bottom τ = 0.25 percentile.


$$\begin{array}{l} L=\left\{c|{\bar{F}}^{\left(c\right)}\leq P_{25}\left(\bar{F}\right)\right\}. \end{array}$$ (3)

In the current scenario with seven maize disease classes in CCMT, selecting the bottom 25% is going to identify one to two classes. Such that there is a targeted improvement without disrupting overall performance balance among all the classes.

#### Step 2: Weighting based on data proportionality

Consistent with the FedAvg, a data-proportional weight is computed for each client as given in (4):


$$\begin{array}{l} w_{i}^{data}=\frac{n_{i}}{\sum_{j=1}^{K}n_{j}}, \end{array}$$ (4)

where *n*_*i*_ represents the number of training samples at client *i*. Clients with a large amount of data contribute more significantly, which gives a high statistical reliability of their updates.

#### Step 3: Performance-based weighting

To better reflect how well each client handles difficult disease categories, we calculate a performance score for every client as given in (5),


$$\begin{array}{l} p_{i}=\{\begin{array}{ll} 0.4\cdot f_{i}^{low}+0.6\cdot f_{i}^{overall}, & \text{if client has samples in }L\\ f_{i}^{overall}, & \text{otherwise} \end{array} \end{array}$$ (5)

Here $f_{i}^{low}$ is the client's average F1-score on the disease classes that are generally hard to classify and that the client actually has data for, and $f_{i}^{overall}$ is the average F1-score across all its available classes.

Clients are evaluated only on disease classes for which they possess training samples ($F_{i}^{\left(c\right)}>0$). This prevents penalizing farms that do not have certain crop diseases.

The 40%–60% weighting maintains balances between specialization and overall model reliability. While expertise on weak classes is rewarded (40%), the overall reliability remains the dominant (60%), preventing over-specialized clients from disproportionately influencing aggregation.

A temperature-scaled softmax is applied to turn these performance scores into usable weights as given in (6):


$$\begin{array}{l} w_{i}^{perf}=\frac{\exp\left(p_{i}/T\right)}{\sum_{j=1}^{K}\exp\left(p_{j}/T\right)}, \end{array}$$ (6)

where *T* = 2.0 controls smoothness.Using a higher temperature spreads the weights more evenly, ensuring that a single top-performing client doesn't overpower the rest.

#### Step 4: Combined weighting

Data-based and performance-based weights are merged as given in (7):


$$\begin{array}{l} w_{i}=\alpha \cdot w_{i}^{data}+\left(1-\alpha \right)\cdot w_{i}^{perf}, \end{array}$$ (7)

where α = 0.7. This approach assigns greater importance to dataset size (70%) while incorporating a 30% contribution from performance-based weighting. Empirical evaluations indicated that this ratio provided the best trade-off between stability and adaptive specialization.

#### Step 5: Momentum smoothing

Exponential moving average smoothing is applied To prevent fluctuations between rounds as given in (8),


$$\begin{array}{l} w_{i}^{\left(r\right)}=\left(1-\beta \right)w_{i}^{\left(r\right)}+\beta w_{i}^{\left(r-1\right)}, \end{array}$$ (8)

where β = 0.3. This stabilizes training by ensuring gradual transitions in aggregation weights.

#### Step 6: Minimum weight constraint

A lower bound of *w*_*min*_ = 0.05 is enforced:


$$\begin{array}{l} w_{i}\leftarrow \max\left(w_{i},w_{min}\right). \end{array}$$ (9)

With 10 clients, equal weighting would be 0.10. A 5% floor ensures no client is excluded entirely while still allowing adaptive prioritization.

#### Step 7: Final normalization

Finally, weights are normalized as given in (10):


$$\begin{array}{l} w_{i}\leftarrow \frac{w_{i}}{\sum_{j=1}^{K}w_{j}}. \end{array}$$ (10)

#### Hyperparameter configuration

Table 7 summarizes the hyperparameters used in AdaClass.

**Table 7:** AdaClass hyperparameters and design rationale.

Parameter	Value	Rationale
*r*_*warmup*_	3	enables stabilization before adaptive weighting
α	0.7	Balances data reliability (70%) and performance expertise (30%)
τ	0.25	Targets bottom 25% of classes (1–2 diseases)
β	0.3	Regualtes smoothing across rounds
*w*_*min*_	0.05	Prevents complete client exclusion
*T*	2.0	Produces smoother performance-based distribution

#### Computational complexity

AdaClass exhibits a computational complexity O(KC) where *K* is the number of clients and *C* is the number of disease classes. This is the same as FedAvg's aggregation step. The additional operations such as computing F1 statistics, identifying low-performing classes and momentum smoothing add negligible overhead compared to model training.

#### Implementation details

For full reproducibility:

- **Warmup rounds:** Fixed to three rounds, regardless of total training rounds
- **F1 computation:** We use scikit-learn's f1_score with zero_division = 0 to handle cases where clients have no samples for a class
- **Softmax implementation:** A standard PyTorch/NumPy softmax function is applied, incorporating temperature scaling prior to exponentiation
- **Momentum initialization:** In the first round, previous weights (**w**_*prev*_) are undefined; therefore, momentum is applied starting from the second round onward.

## Experimental evaluation protocol

To evaluate the effectiveness of the proposed federated learning framework for crop disease detection, a diverse set of quantitative metrics and statistical evaluation methods are used. This section describes the classification metrics used to measure predictive performance, the receiver operating characteristic (ROC) analysis and the procedures employed to analyze computational efficiency. Together, these components provide a good understanding of both model accuracy and practical deployability.

###  Classification performance metrics

For multi-class classification problems with *C* classes, we define *TP*_*i*_, *TN*_*i*_, *FP*_*i*_, and *FN*_*i*_ as the true positives, true negatives, false positives, and false negatives for class *i*, respectively. These quantities form the basis for computing all subsequent evaluation metrics.

**Accuracy** measures the overall proportion of correctly classified samples across all classes as given in (11),


$$\begin{array}{l} \text{Accuracy}=\frac{\sum_{i=1}^{C}TP_{i}}{\sum_{i=1}^{C}\left(TP_{i}+FP_{i}+TN_{i}+FN_{i}\right)} \end{array}$$ (11)

**Precision** measures the ratio of true positives to all predicted positives. To account for varying class frequencies, we compute Weighted Precision, which averages class-level precision scores according to each class's support (i.e., number of true samples) as given in (12) and (13):


$$\begin{array}{lll} {\text{Precision}}_{i} & = & \frac{TP_{i}}{TP_{i}+FP_{i}} \end{array}$$ (12)

$$\begin{array}{lll} \text{Weighted Precision} & = & \frac{\sum_{i=1}^{C}{\text{Precision}}_{i}\times {\text{Support}}_{i}}{\sum_{i=1}^{C}{\text{Support}}_{i}} \end{array}$$ (13)

where Support_*i*_ represents the number of actual occurrences of class *i* in the dataset.

**Recall (sensitivity)** is the proportion of actual positive cases that the model successfully identifies as given in (14),


$$\begin{array}{l} {\text{Recall}}_{i}=\frac{TP_{i}}{TP_{i}+FN_{i}} \end{array}$$ (14)

Similarly, Weighted Recall aggregates these scores based on class support as given in (15),


$$\begin{array}{l} \text{Weighted Recall}=\frac{\overset{C}{\underset{i=1}{\sum }}{\text{Recall}}_{i}\times {\text{Support}}_{i}}{\overset{C}{\underset{i=1}{\sum }}{\text{Support}}_{i}} \end{array}$$ (15)

**F1-score** combines precision and recall into a single metric by taking their harmonic mean as given in (16). This is particularly useful in settings with class imbalance—an issue commonly found in agricultural disease datasets:


$$\begin{array}{lll} {\text{F1}}_{i} & = & 2\times \frac{{\text{Precision}}_{i}\times {\text{Recall}}_{i}}{{\text{Precision}}_{i}+{\text{Recall}}_{i}} \end{array}$$ (16)

$$\begin{array}{lll} \text{Weighted F1} & = & \frac{\sum_{i=1}^{C}{\text{F1}}_{i}\times {\text{Support}}_{i}}{\sum_{i=1}^{C}{\text{Support}}_{i}} \end{array}$$ (17)

Using weighted averages as given in (17) ensures that classes with more samples have a proportional impact on the overall evaluation. This approach is especially important for agricultural disease detection, where common diseases naturally appear more frequently than rare ones, and unweighted averaging could obscure real-world performance differences (McMahan et al., 2017).

**Cohen's d** is a standardized effect size for measuring the difference between two paired methods, capturing the magnitude of improvement as given in (18).


$$\begin{array}{l} d=\frac{\bar{d}}{s_{d}} \end{array}$$ (18)

where $\bar{d}$ is the mean of the paired differences as mentioned in (19):


$$\begin{array}{l} \bar{d}=\frac{1}{n}\overset{n}{\underset{i=1}{\sum }}\left(x_{i}-y_{i}\right) \end{array}$$ (19)

and *s*_*d*_ is the standard deviation of these differences as given in (20):


$$\begin{array}{l} s_{d}=\sqrt{\frac{1}{n-1}\overset{n}{\underset{i=1}{\sum }}{\left(d_{i}-\bar{d}\right)}^{2}} \end{array}$$ (20)

Here, *x*_*i*_ and *y*_*i*_ represent the performance of two methods (e.g., AdaClass and a baseline) under the same experimental condition (seed *i*). Cohen's d provides a scale independent measure of effect size, where higher values indicate stronger.

**Rank-biserial correlation** (*r*_*rb*_) is a non-parametric effect size as given in (21) used with the Wilcoxon signed-rank test to measure consistency of differences:


$$\begin{array}{l} r_{rb}=\frac{W^{+}-W^{-}}{W^{+}+W^{-}} \end{array}$$ (21)

where *W*^+^ and *W*^−^ are the sums of ranks for positive and negative differences, respectively, computed after ranking absolute differences.

The value of *r*_*rb*_ ranges from −1 to 1, where higher values indicate more consistent improvement of one method over another.

###  Receiver operating characteristic analysis

Receiver Operating Characteristic (ROC) curve analysis evaluates the model's ability to distinguish between classes at different decision thresholds. In the multi-class setting, micro-averaging is used, which combines the predictions from all classes to generate a single overall ROC curve and AUC value. This method treats every prediction equally, making it suitable for imbalanced datasets. Otherwise, per-class ROC measurements may be misleading.

The ROC curve plots the True Positive Rate (TPR) against the False Positive Rate (FPR) as given in (22) and (23):


$$\begin{array}{lll} \text{TPR} & = & \frac{\sum_{i=1}^{C}TP_{i}}{\sum_{i=1}^{C}\left(TP_{i}+FN_{i}\right)} \end{array}$$ (22)

$$\begin{array}{lll} \text{FPR} & = & \frac{\sum_{i=1}^{C}FP_{i}}{\sum_{i=1}^{C}\left(FP_{i}+TN_{i}\right)} \end{array}$$ (23)

The Area Under the Curve (AUC) provides a single scalar value summarizing classifier performance as defined in (24):


$$\begin{array}{l} \text{AUC}=\int_{0}^{1}\text{TPR}\left(t\right)d\left(\text{FPR}\left(t\right)\right) \end{array}$$ (24)

where *t* represents the classification threshold. AUC values approaching 1.0 indicate superior discriminative capability, while values near 0.5 suggest performance equivalent to random classification.

###  Computational efficiency assessment

A rigorous computational efficiency analysis has been carried out to evaluate model suitability for edge deployment in agricultural settings. Our assessment methodology incorporates statistical analysis of inference performance under controlled conditions.

#### Inference time measurement protocol

A standardized inference time measurement protocol has been implemented that accounts for performance variability and system warm-up effects. Algorithm 2 outlines our statistical analysis procedure for inference latency.

Algorithm 2 Inference time statistical analysis protocol. 
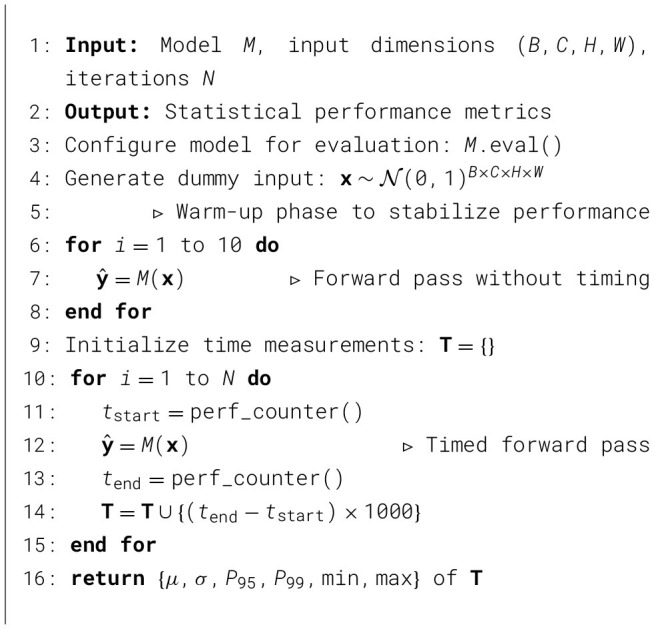

#### Statistical performance metrics

The computational evaluation incorporates multiple statistical measures to assess inference performance:

- **Mean latency** (μ): Average inference time across all measurements.
- **Standard deviation** (σ): The Variability in inference performance.
- **95th percentile** (*P*_95_): Upper bound for 95% of inference times.
- **99th percentile** (*P*_99_): Worst-case performance under normal conditions.

The 95th percentile metric is particularly valuable for real-time agricultural applications, as it provides a reliability threshold for deployment planning. Models with *P*_95_ < 100 ms are generally considered suitable for interactive agricultural monitoring applications (Warden and Situnayake, 2019).

#### Model size analysis

We quantify memory requirements through comprehensive model size analysis as given in (25):


$$\begin{array}{l} \text{Model Size (MB)}=\frac{\begin{array}{c} \sum_{p\in \text{Parameters}}|p|\times \text{sizeof}\left(p\right)\\ +\sum_{b\in \text{Buffers}}|b|\times \text{sizeof}\left(b\right) \end{array}}{1024^{2}} \end{array}$$ (25)

This metric directly correlates with deployment feasibility on resource-constrained edge devices commonly used in agricultural settings (Warden and Situnayake, 2019). The proposed model achieve deployment efficiency targets while maintaining classification accuracy suitable for practical agricultural use.

###  Federated learning evaluation metrics

To assess the effectiveness of the proposed AdaClass strategy, several federated learning–specific evaluation metrics are incorporated in addition to standard classification measures.

- **Per-class F1 convergence:** We track how the F1-scores of individual disease classes especially the more challenging ones evolve over every communication round. This helps verify whether the dynamic aggregation mechanism successfully prioritizes and improves performance on difficult classes.
- **Client contribution weights:** We analyze how the aggregation weights assigned to each client change throughout the training process. This reveals how the strategy adapts to differences in client expertise, data quality, and class distributions, providing insight into the fairness and adaptivity of the weighting scheme.
- **Communication efficiency:** Rounds required to achieve target accuracy thresholds, validating that intelligent aggregation reduces communication overhead compared to standard FedAvg (McMahan et al., 2017).

These federated learning metrics enable comprehensive assessment of our approach's ability to balance privacy preservation by avoiding raw data sharing, with collaborative model improvement across heterogeneous agricultural data distributions (Kairouz and McMahan, 2021).

## Results and discussions

###  CCMT dataset results

Table 8 presents performance comparison of different models on the CCMT maize dataset. The results demonstrate that incorporating MobileViT transformer blocks consistently improves performance over baseline CNN models. Among the baseline architectures, ResNet-50 achieved the a accuracy of 92.4%, while MobileNetV2 baseline showed lower performance at 87.77%.

**Table 8:** CCMT maize dataset: model performance comparison.

Model	Accuracy	Weighted F1	Weighted precision	Weighted recall
MobileNetV2 (Base)	87.77%	0.877	0.877	0.877
ResNet-50 (Base)	92.4%	0.92	0.923	0.92
MobileNetV2+MobileViT	91.4%	0.91	0.912	0.914
EfficientNet-B0+MobileViT	92.5%	0.921	0.924	0.925
ResNet-34+MobileViT	90.01%	0.900	0.900	0.900

With hybrid CNN-Transformer architectures, accuracy improved across all models. EfficientNet-B0 combined with MobileViT achieved the best results with 92.5% accuracy and balanced F1-scores which is comparable with Resnet-50. MobileNetV2 combined with MobileViT follows closely, achieving an accuracy of 91.4%. These improvements are also reflected in the weighted F1-scores, indicating that hybrid architectures are better equipped to handle challenging disease classes compared to standard baseline models.

**Figure 3:**
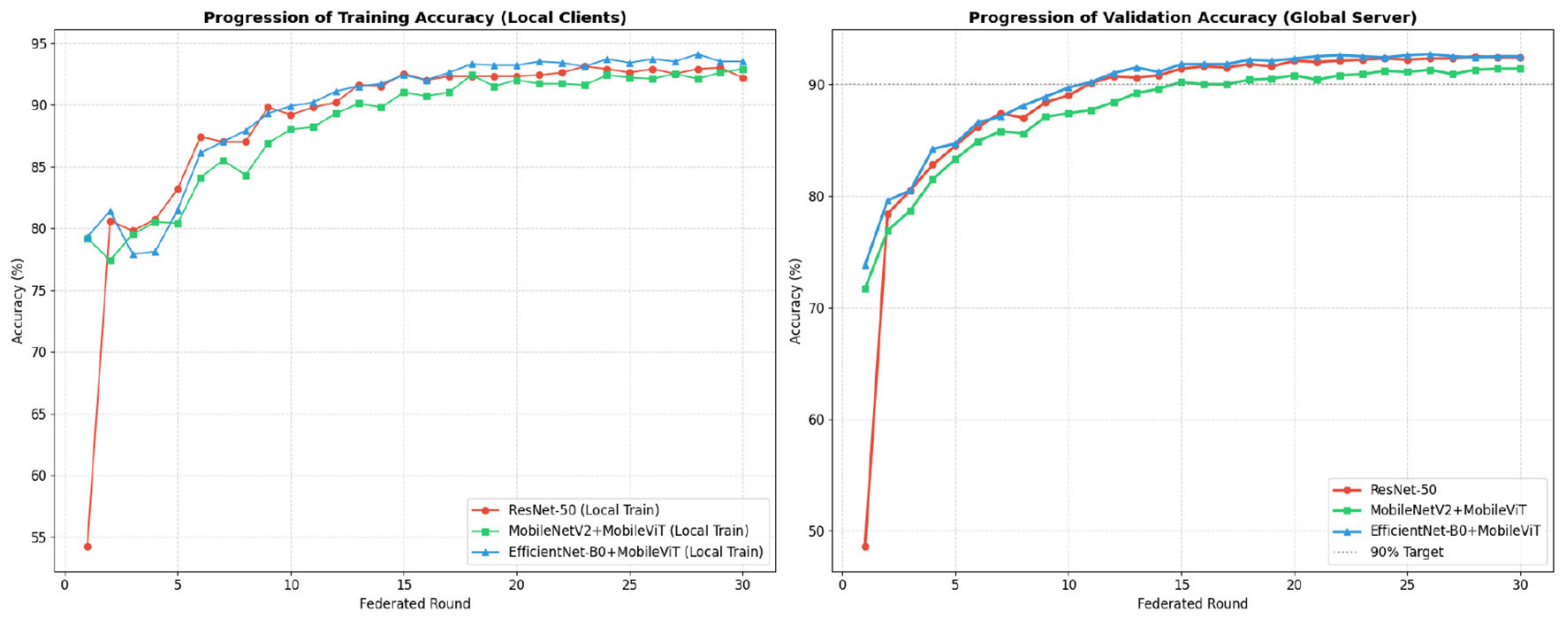
Model accuracy improvement across communication rounds for the CCMT maize dataset.

Figure 3 illustrates the convergence of different models on the CCMT dataset with 3 clients across 30 communication rounds. Resnet50 shows instability at initial rounds which we account to its high parameter count making it sensitive toward non-IID distribution before sufficient aggregation takes place However, hybrid EfficientNet-B0 steadily improves and shows convergence, comparable to the baseline Resnet-50 model, with both models converging to approximately 92% accuracy. MobileNetV2 shows slower initial convergence but demonstrates consistent improvement, reaching approximately 91% accuracy by the final round.

The experimentation is done on 3 clients to validate proposed architecture and algorithm under controlled with reduced heterogeneity of disease classes among clients. This leads to isolation of the model architecture contributions from federated complexity.

The small gap present between training and testing accuracy across the models suggests that there is limited overfitting under the federated learning setup.

###  PlantVillage dataset results

Table 9 shows the federated learning results on the PlantVillage dataset. The hybrid EfficientNet-B0 with MobileViT achieved 99.32% accuracy with balanced precision, recall, and F1-scores. ResNet-50 with CBAM (using standard FedAvg) achieved slightly higher accuracy at 99.66%, while hybrid MobileNetV2 with MobileViT reached 99.17%. These results demonstrate the effectiveness of hybrid CNN-Transformer architectures for distributed agricultural disease detection, with EfficientNet-B0 providing excellent overall performance.

**Table 9:** PlantVillage dataset: model performance comparison.

Model	Accuracy	Wtd F1	Wtd Prec	Wtd Rec
ResNet-50+CBAM (FedAvg)	99.66%	0.9966	0.9977	0.9966
EfficientNet-B0+MobileViT	99.32%	0.994	0.994	0.994
MobileNetV2+MobileViT	99.17%	0.9917	0.9917	0.9917

**Figure 4:**
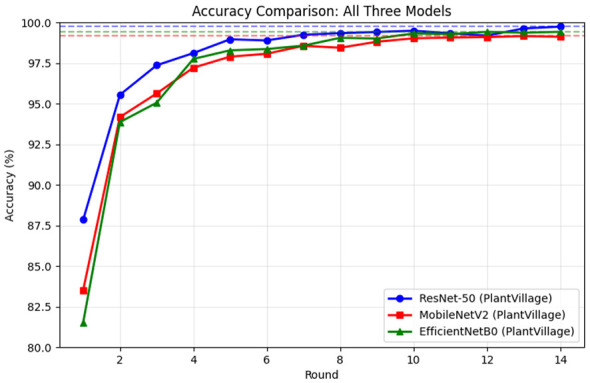
Model accuracy convergence on the PlantVillage dataset across communication rounds.

Figure 4 presents the convergence behavior on the PlantVillage dataset, demonstrating rapid improvement across communication rounds. All models achieve very high accuracy, with minimal differences between approaches by the final round, indicating that the dataset's cleaner, laboratory-controlled conditions enable all models to reach near-optimal performance. The convergence rate is substantially faster than on CCMT, reflecting the reduced complexity of laboratory-controlled imagery.

###  Confusion matrix analysis

**Figure 5:**
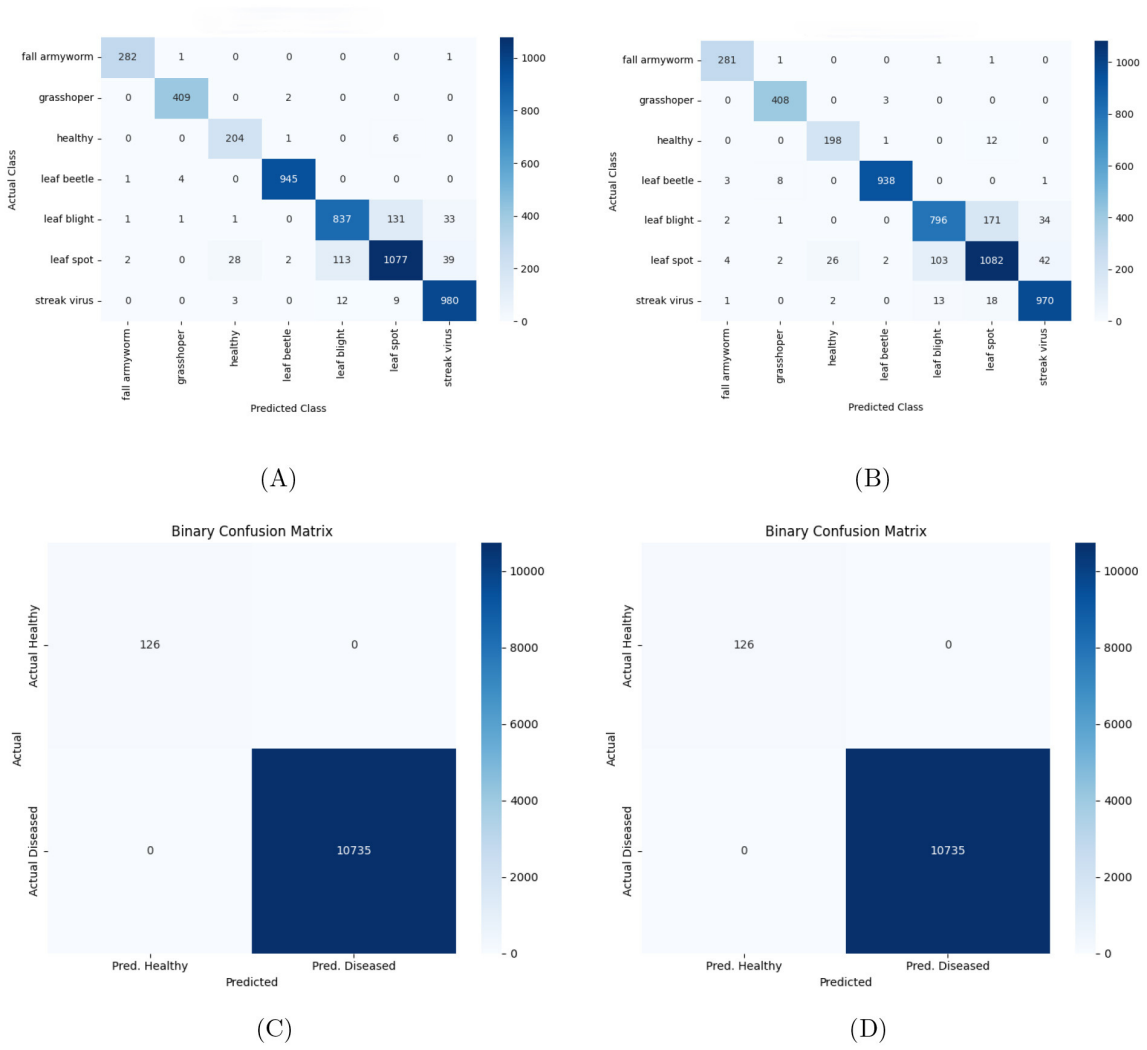
Confusion matrices for hybrid models on plant disease classification across datasets. **(A)** EfficientNet-B0 on CCMT dataset, **(B)** EfficientNet-B0 on PlantVillage dataset, **(C)** MobileNetV2 on CCMT dataset, and **(D)** MobileNetV2 on PlantVillage dataset.

Figure 5 shows the discriminative ability of both the hybrid models EfficientNetB0 and MobileNetV2 across both datasets. The hybrid models perform exceptionally well, with EfficientNetB0 showing a strong ability to differentiate all the 7 disease classes on maize subset of data. MobileNetV2 also shows a strong performance but has slightly more misclassifications when compared to EfficientNetB0 for the 3-client setup. The diagonal in the confusion matrix proves that both the hybrid models learn to differentiate features and separate disease classes reliably.

###  Receiver operating characteristic analysis

**Figure 6:**
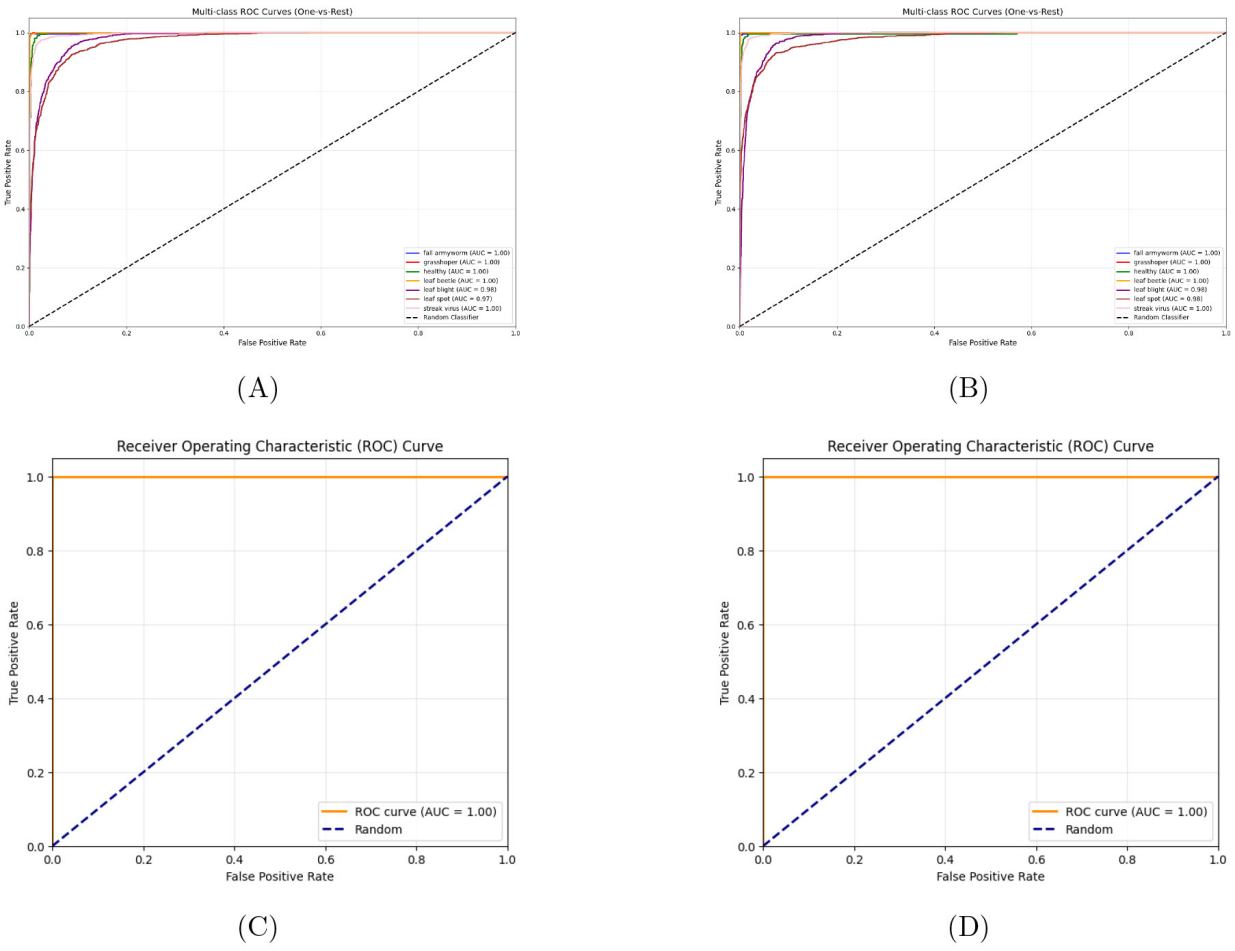
ROC curves and AUC scores for hybrid model evaluation on plant disease classification. **(A)** MobileNetV2 on CCMT maize dataset (AUC >0.98), **(B)** EfficientNet-B0 on CCMT maize dataset (AUC >0.98), **(C)** MobileNetV2 on PlantVillage dataset (AUC >0.99), and **(D)** EfficientNet-B0 on PlantVillage dataset (AUC >0.99).

Figure 6 shows a strong discriminative ability of both the hybrid models EfficientNetB0 and MobileNetV2 across both datasets. The hybrid models MobileNetV2 and EfficientNetB0 both show an AUC value of above 0.98 which demonstrates a strong ability to distinguish between the 7 disease classes in CCMT maize subset. In plantVilage dataset both the models score above AUC value of 0.99 between healthy and disease classes. This demonstrates that both the models have an exceptional ability to distinguish crop disease classes across both controlled and field-captured.

###  Ablation study: baseline vs. hybrid architecture

To validate the contribution of each architectural component, we conducted ablation studies comparing baseline CNN models with the proposed hybrid models. This is shown in Table 10 which demonstrates the cumulative benefit of our design choices.

**Table 10:** Ablation study: architecture component contributions.

Architecture	Accuracy	Improvement	F1-score
MobileNetV2+Attention	87.77%	—	0.8777
MobileNetV2+MobileViT+Attention	91.4%	+3.63%	0.915
EfficientNet-B0+CBAM+ESA	~89.5%	—	~0.8950
EfficientNet-B0+MobileViT+Attention	92.5%	+3.0%	0.925

Starting with baseline MobileNetV2, which achieved 87.77% accuracy on the CCMT dataset, we progressively added MobileViT blocks along with attention mechanisms (CBAM and ESA). The incorporation of MobileViT blocks provided global feature modeling capability, while CBAM and ESA focused attention on discriminative features and spatial regions containing disease symptoms. The resulting MobileNetV2+MobileViT+Attention architecture achieved 91.4% accuracy, representing a 3.25% improvement over the baseline.

Similarly, for EfficientNet-B0, adding MobileViT and attention mechanisms increased accuracy from approximately 89.5% to 92.5%, a 3% improvement. These improvements validate that the hybrid CNN-Transformer approach with attention mechanisms provides meaningful benefits for crop disease detection, particularly for challenging disease classes in federated learning settings.

###  Ablation study: proposed vs. existing aggregation techniques

To evaluate how well the proposed Adaptive Class-Aware Aggregation (AdaClass) performs, we compare it against three widely used federated optimization methods—FedAvg, FedProx, and FedDyn—using both the CCMT Maize and CCMT Tomato datasets. All experiments are conducted under the same conditions, including identical client partitions, the same number of communication rounds, consistent optimizer settings, and the same model architecture. No additional tuning or enhancement techniques are introduced, ensuring a fair comparison that isolates the impact of the aggregation method itself.

#### Overall performance comparison

Tables 11, 12 summarize the overall performance of the four methods on the CCMT Maize and CCMT Tomato datasets respectively.

**Table 11:** Overall performance comparison on CCMT maize dataset.

Method	Accuracy (%)	Macro F1	Weighted F1
FedAvg	90.43 ± 0.80	0.919 ± 0.010	0.904 ± 0.009
FedProx	90.49 ± 0.68	0.919 ± 0.009	0.904 ± 0.007
FedDyn	90.07 ± 0.87	0.916 ± 0.013	0.900 ± 0.009
AdaClass (proposed)	90.79 ± 0.57	0.921 ± 0.008	0.906 ± 0.006

**Table 12:** Overall performance comparison on CCMT tomato dataset.

Method	Accuracy (%)	Macro F1	Weighted F1
FedAvg	92.74 ± 1.46	0.920 ± 0.019	0.926 ± 0.016
FedProx	90.70 ± 1.34	0.900 ± 0.017	0.906 ± 0.014
FedDyn	91.19 ± 1.36	0.902 ± 0.018	0.910 ± 0.015
AdaClass (proposed)	92.98 ± 1.13	0.924 ± 0.014	0.929 ± 0.012

On Maize, the proposed AdaClass method achieves an overall accuracy of 90.79%, outperforming FedDyn by 0.72%, FedAvg by 0.36%, and FedProx by 0.30%. FedAvg and FedProx tie on Macro F1 at 0.919, while AdaClass edges ahead at 0.921, followed by FedDyn at 0.916. In terms of Weighted F1, AdaClass again achieves the highest value (0.906), compared to FedAvg and FedProx (0.904) and FedDyn (0.900).

AdaClass also exhibits lower standard deviation across all three metrics. On Maize, the accuracy standard deviation is ±0.57, compared to FedAvg (±0.80), FedProx (±0.68), and FedDyn (±0.87).

In addition to the mean and standard deviations, we also calculated 95% confidence intervals to assess statistical reliability across runs. For example, AdaClass achieves 90.79 ± 0.57 (CI ≈ ± 0.50), compared to FedAvg at 90.43 ± 0.80 (CI ≈ ±0.70) on the maize dataset. A similar trend is observed for both FedDyn and FedProx. This lower variance indicates that AdaClass converges more reliably across different data splits and delivers consistent performance, particularly for minority disease classes.

As the current experimentation was performed on a limited no.of runs (*n* = 5 seeds), we report effect size measures rather than p-values, as that is unreliable at this sample size. The effect size analysis adds to the FedDyn comparison, where Cohen's *d* = 0.869 (large) and *r*_*rb*_ = 1.00 indicates that AdaClass consistently outperformed FedDyn on all five seeds. Against FedAvg and FedProx, effect sizes are medium (*d* = 0.514, *d* = 0.586; *r*_*rb*_ = 0.80), with AdaClass ranking higher on 4 of 5 seeds in both cases.

In agricultural deployment scenarios where rare disease detection is critical, a method that reliably identifies all disease types is more valuable than one that maximizes average accuracy at the expense of class balance (Shafay et al., 2025).

#### Per-class performance analysis

Tables 13, 14 break down the F1-score for each disease class across all four methods on the CCMT Maize and CCMT Tomato datasets respectively, averaged over three random seeds.

**Table 13:** Per-class F1-score comparison across federated aggregation methods on the CCMT Maize dataset.

Disease class	FedAvg	FedProx	FedDyn	AdaClass
Fall armyworm	0.981 ± 0.006	0.980 ± 0.006	0.979 ± 0.006	0.981 ± 0.008
Grasshopper	0.995 ± 0.002	0.996 ± 0.001	0.996 ± 0.002	0.995 ± 0.002
Healthy^*^	0.897 ± 0.039	0.896 ± 0.042	0.886 ± 0.053	0.898 ± 0.031
Leaf beetle	0.993 ± 0.004	0.991 ± 0.002	0.991 ± 0.003	0.992 ± 0.003
Leaf blight^*^	0.836 ± 0.006	0.832 ± 0.009	0.824 ± 0.010	0.836 ± 0.003
Leaf spot^*^	0.807 ± 0.023	0.813 ± 0.012	0.805 ± 0.015	0.812 ± 0.019
Streak virus	0.928 ± 0.021	0.928 ± 0.012	0.926 ± 0.014	0.935 ± 0.010
Macro F1	0.919 ± 0.010	0.919 ± 0.009	0.916 ± 0.013	0.921 ± 0.008

Values reported as mean ± std over five seeds (42, 123, 7, 0, 99). [*] Challenging class flagged by AdaClass struggling-class detector. AdaClass achieves the best or joint-best F1 on 4 of 7 classes and the lowest variance on Leaf Blight (±0.003), Healthy (±0.031 vs. ±0.053 for FedDyn), and Streak Virus (±0.010).

**Table 14:** Per-class F1-score comparison across federated aggregation methods on the CCMT Tomato dataset.

Disease class	FedAvg	FedProx	FedDyn	AdaClass
Healthy	0.977 ± 0.005	0.969 ± 0.003	0.965 ± 0.002	0.980 ± 0.002
Leaf blight^*^	0.905 ± 0.029	0.879 ± 0.020	0.896 ± 0.013	0.913 ± 0.020
Leaf curl	0.925 ± 0.008	0.905 ± 0.014	0.904 ± 0.006	0.924 ± 0.006
Septoria leaf spot	0.952 ± 0.004	0.933 ± 0.005	0.937 ± 0.007	0.950 ± 0.004
Verticillium wilt^*^	0.841 ± 0.063	0.813 ± 0.048	0.809 ± 0.073	0.855 ± 0.040
Macro F1	0.920 ± 0.019	0.900 ± 0.017	0.902 ± 0.018	0.924 ± 0.014

Values reported as mean ± std over three seeds (123, 0, 7). [*] Challenging class flagged by AdaClass struggling-class detector. AdaClass achieves the best F1 on 3 of 5 classes and the lowest variance on Verticillium Wilt (±0.040 vs. ±0.073 for FedDyn), the hardest class in the Tomato subset.

Although these improvements are modest, the consistency of these trends across multiple classes and crops suggests that the gains are systematic rather than being incidental.

Classes such as Grasshopper and Fall Armyworm in Maize and Leaf Curl and Septoria Leaf Spot in Tomato exhibit similar performance across all four methods. These classes were well represented across the clients. Challenging classes like Healthy, Leaf Spot and Leaf Blight in Maize and Verticillium Wilt and Leaf Blight in Tomato provide distinct and meaningful results.

In the Maize Healthy class FedAvg achieves 0.897 on the other hand, FedDyn performs a bit poorly with F1-score 0.886 and FedProx also struggles at 0.896. AdaClass achieves a slightly higher F1-score 0.898 and a much smaller variation of ±0.031 compared to FedDyn's ±0.053, showing that AdaClass shows improved stability and it is consistent across different data splits. Coming to Maize Leaf Blight, FedAvg and AdaClass reach a mean performance of 0.836 but AdaClass maintains a smaller variation of ±0.003, indicating a stable convergence. On Maize Leaf Spot, all methods struggle due to visual overlap with early-stage Leaf Blight, though AdaClass tops with the best mean of 0.812 while FedDyn lags at 0.805.

In the Tomato Verticillium Wilt class FedAvg achieves 0.841 on the other hand, FedDyn performs a bit poorly with F1-score 0.809 and FedProx also struggles at 0.813. AdaClass tops with 0.855 and a much smaller variation of ±0.040 compared to FedDyn's ±0.073, showing that AdaClass shows improved stability. Coming to Tomato Leaf Blight, FedAvg stays competitive at 0.905, while AdaClass reaches 0.913 with a variation of ±0.020. The higher F1-score of FedAvg on Septoria Leaf Spot and Leaf Curl in Tomato and Leaf Blight in Maize can be attributed to its data proportional weighting. This favors clients with more samples from these dominant classes in these particular splits.

AdaClass demonstrates marginal improvements, it also displays more stability and consistent behavior, especially on challenging classes by identifying them and adaptively emphasizes those clients that perform well on them. As a result the global model receives reliable updates for challenging classes, regardless of the heterogenous data distributions across clients.

On Maize, AdaClass demonstrates a slightly higher Macro F1 of 0.921 with reduced variability (±0.008) compared to FedAvg and FedProx at 0.919 ± 0.010 and 0.919 ± 0.009 and FedDyn at 0.916 ± 0.013. On Tomato, AdaClass again shows a slightly higher Macro F1 of 0.924 with the lowest standard deviation (±0.014) compared to FedAvg at 0.920 ± 0.019, FedProx at 0.900 ± 0.017, and FedDyn at 0.902 ± 0.018.

In an agricultural setting, failing to classify a crop disease class like Verticillium Wilt or a visually difficult one like Leaf Blight can have an impact on crop health management. The results suggest that AdaClass improves challenging classes without compromising the performance on the rest, and the federated model becomes more dependable for real-world crop disease detection deployment across multiple crops.

###  Sensitivity analysis of hyperparameters

To understand how sensitive the AdaClass is to the value of α, we evaluated the following four α values 0, 0.25, 0.5, 0.75, and 1 (FedAvg) by using a single seed. The results shown in Table 15. show that the middle range alpha values produce a similar accuracy. When the value of α is 1, AdaClass basically becomes FedAvg and produces a Macro F1-score of 0.914 and a weighted F1 0.906, which is the lowest among all the other values. This says that removing the performance based weighting does affect the model's ability to handle the disease classes fairly. On the other hand, when the value of alpha is set to 0, algorithm completely relies on performance and ignores the data size of each client. This allows noisy and data sparse clients influence the global model which also reduces the accuracy and F1-score.

**Table 15:** Sensitivity analysis of the mixing parameter α in AdaClass at seed value 7.

α	Accuracy (%)	Macro F1	Weighted F1
0.00	90.95	0.924	0.908
0.25	91.12	0.928	0.910
0.50	90.93	0.927	0.909
**0.70**	**91.24**	0.931	0.914
1.00 (FedAvg)	90.89	0.914	0.906

Bold values indicate the best result per column.

The configurations between these two extremes perform consistently well, indicatinag that AdaClass is robust to fine-grained tuning and will work reasonably across a range of setting. Among all the evaluated values α = 0.7 provides the best result across all the evaluation metrics and is chosen as the default value. Table 11 provides a stronger evidence with three seed comparisons where AdaClass has reduced variance compared to FedAvg (±0.57 vs. ±080), which shows that adaptive weighting consistently produces more reliable results across different data conditions.

###  Convergence and aggregation stability analysis

To further evaluate the robustness of the proposed AdaClass strategy, we analyze both performance convergence and the evolution of client aggregation weights across federated rounds.

#### F1-score convergence

**Figure 7:**
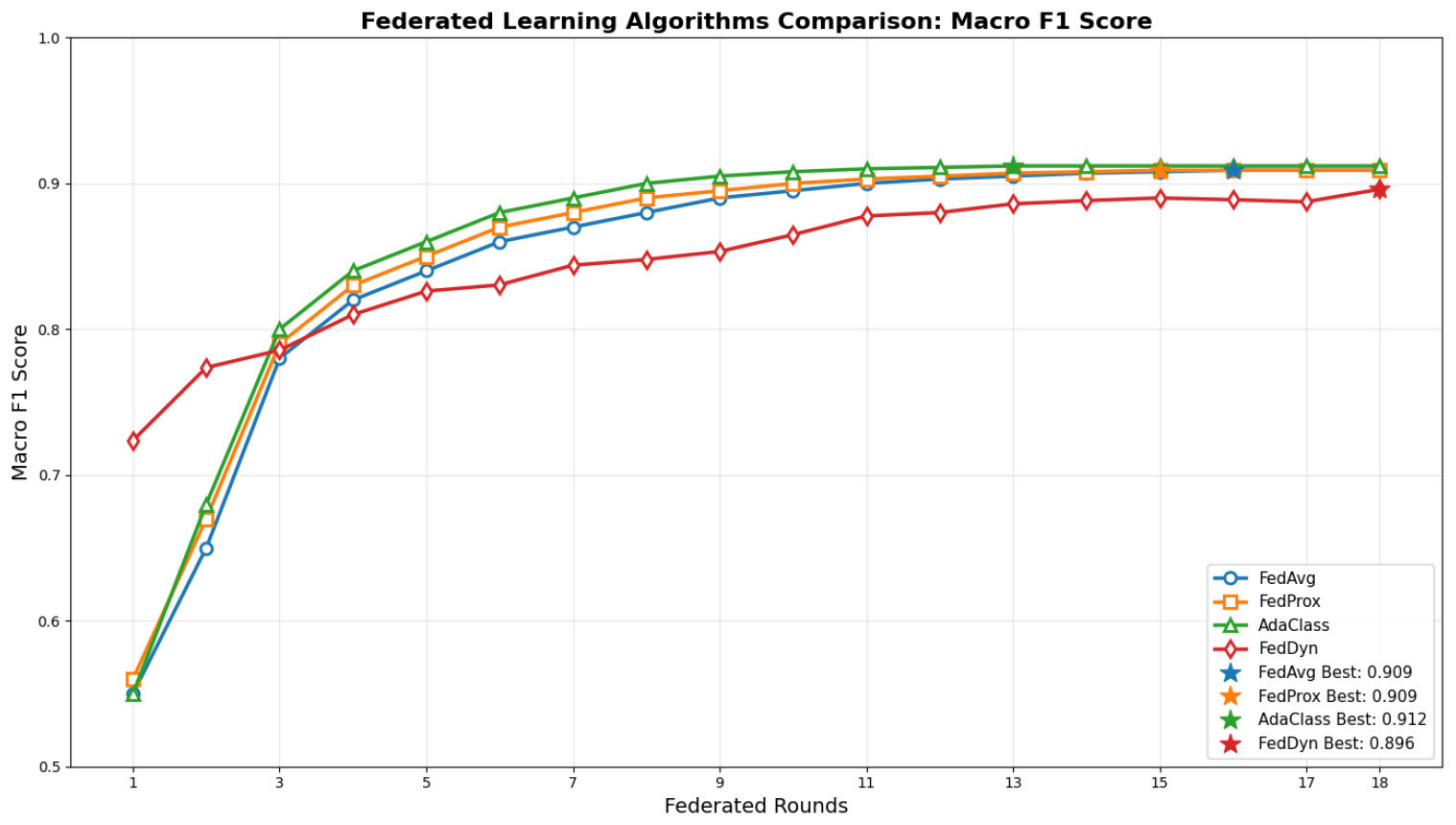
Macro-F1 convergence across 18 federated rounds for FedAvg, FedProx, FedDyn, and AdaClass on Maize. The proposed method maintains stable convergence while achieving improved balanced performance.

Figure 7 shows Macro F1 progression across 18 federated rounds for FedAvg, FedProx, FedDyn, and the proposed AdaClass method shows a stable and meaningful convergence patterns throughout the experimental evaluation. Moreover, the important results may suggest that all 4 methods demonstrate stable convergence, showing that existing algorithms achieve gradual improvement and plateau in later rounds. However, findings indicate AdaClass continues to improve steadily after a 3-round warmup phase. Thus, evidence shows adaptive aggregation does not introduce instability or oscillatory behavior. By round 8 AdaClass achieves a Macro F1 score of 0.9, whereas the exisintg methods FedAvg and FedProx reach a similiar performance only after round 10–12, which shows faster and reliable convergence of AdaClass. Additionally, results indicate that the smooth trajectory of the adaptive mechanism integrates seamlessly with the optimization process.

#### Client weight evolution

**Figure 8:**
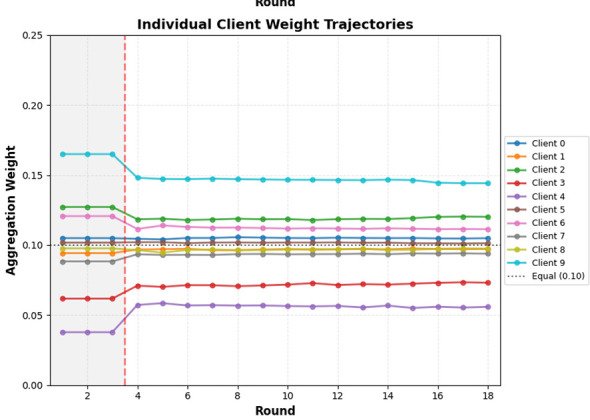
Evolution of client weights using AdaClass aggregation across 18 rounds.

Figure 8 shows update of weights in the proposed AdaClass aggregation algorithm across 18 rounds. The trend in the graph shows that methodological choices that are chosen influence the observed result. The dashed vertical line is the shift from warmup phase where FedAvg is used to adaptive aggregation, demonstrating a meaningful structural change.

###  Computational efficiency analysis

To evaluate suitability for edge device deployment, we conducted comprehensive analysis of model size and inference latency. Table 16 presents computational efficiency metrics for all models.

**Table 16:** Computational efficiency analysis.

Model	Size (MB)	Mean latency (ms)	Std dev (ms)	P95 (ms)
MobileNetV2 hybrid	7.63	41.2	17.4	83.5
EfficientNet-B0 hybrid	18.71	58.2	12.7	81.4
ResNet-50 baseline	89.93	181.2	41.4	260.2

The EfficientNet-B0 hybrid architecture maintained a compact size of 18.71 MB with average CPU inference latency of 58.2 ms (batch size = 1, 224 × 224 input resolution). MobileNetV2 hybrid achieved superior efficiency with only 7.63 MB storage and 41.2 ms mean inference latency, making it particularly suitable for resource-constrained agricultural edge devices. In contrast, ResNet-50 baseline required 89.93 MB storage with significantly higher latency of 181.2 ms, demonstrating the efficiency advantage of lightweight architectures.

The 95th percentile latency metrics are particularly relevant for real-time agricultural monitoring applications. MobileNetV2 hybrid maintained *P*_95_ = 83.5 ms, while EfficientNet-B0 achieved *P*_95_ = 81.4 ms. Both hybrid models satisfy the 100 ms threshold for interactive agricultural monitoring applications. These metrics confirm that our lightweight hybrid architectures provide favorable accuracy-efficiency trade-offs for practical edge-based crop disease detection systems.

These results demonstrate that hybrid CNN-Transformer architectures achieve high classification accuracy while maintaining computational efficiency suitable for practical agricultural deployment scenarios where edge devices operate under strict memory and latency constraints.

## Conclusion

This paper presents AdaClass, a Federated Deep Learning with adaptive weighting method, which focuses on the classes that the global model struggles with each round and pays special attention to the clients that are performing well on those challenging classes. This results in balanced performance across all the classes. The proposed hybrid CNN architecture uses MobileViT, which is combined with two CNN models EfficientNetB0 and MobileNetV2. Both the models are lightweight and MobileNetV2 can process an image with roughly 41.2 milliseconds but takes only 7.63MB and EfficientNet-B0 achieves an accuracy of 92.5% on CCMT, 99.32% on PlantVillage. The key finding of AdaClass is that it does so in a more consistent manner-particularly on disease classes that are rare or visually difficult to separate, where other methods show higher variance across experimental runs. In CCMT, across both the Maize and Tomato crops, AdaClass displays considerably faster convergence and lower variance in Macro F1 compared to the existing aggregation methods. On the struggling classes like the Maize Leaf Blight (±0.003 vs. ±0.012 for FedDyn) and Tomato Verticillium Wilt (±0.040 vs. ±0.073 for FedDyn) the stability gap is consistent with the struggling-class detection mechanism having its intended effect. Across both the crops a consistent and reliable result is obtained with reduced variability which is more practical for agricultural environment.

In the future, this work can be expanded by considering the limitation that the current experimental setups only account for the static data distribution. In the real word, local data distributions vary due to time based on the seasonal changes, environmental and geographical factors. The current approach does not account for this dynamic data distribution. In order to improve the reliable communication, strengthening the federated learning (FL) infrastructure is essential with devices located in remote and geographically diverse regions. Although Federated Learning offers various privacy preserving benefits, through differential privacy one can receive stronger security guarantees (Duchi et al., 2014). Incorporating such mechanism in the future is important as the current framework does not address privacy guarantee.

## Data Availability

The original contributions presented in the study are included in the article/supplementary material, further inquiries can be directed to the corresponding author.
